# Prehospital Large-Vessel Occlusion Stroke Detection Scales: A Pooled Individual Patient Data Analysis of 2 Prospective Cohorts

**DOI:** 10.1212/WNL.0000000000213570

**Published:** 2025-04-08

**Authors:** Luuk Dekker, Jasper D. Daems, Mariam Ali, Martijne H.C. Duvekot, Truc My T. Nguyen, Esmee Venema, Marcel D.J. Durieux, Erik W. van Zwet, Walid Moudrous, Ido R. van den Wijngaard, Henk Kerkhoff, Hester F. Lingsma, Diederik W.J. Dippel, Marieke J.H. Wermer, Bob Roozenbeek, Nyika D. Kruyt, for the LPSS and PRESTO investigators

**Affiliations:** 1 Department of Neurology, Leiden University Medical Center, the Netherlands;; 2 Department of Neurology, Haga Hospital, The Hague, the Netherlands;; 3 Department of Neurology, Erasmus MC University Medical Center, Rotterdam, the Netherlands;; 4 Department of Public Health, Erasmus MC University Medical Center, Rotterdam, the Netherlands;; 5 Department of Neurology, Albert Schweitzer Hospital, Dordrecht, the Netherlands;; 6 Department of Neurology, Amsterdam University Medical Center, the Netherlands;; 7 Department of Emergency Medicine, Erasmus MC University Medical Center, Rotterdam, the Netherlands;; 8 Emergency Medical Services Hollands-Midden, Leiden, the Netherlands;; 9 Department of Medical Statistics, Leiden University Medical Center, the Netherlands;; 10 Department of Neurology, Maasstad Hospital, Rotterdam, the Netherlands;; 11 Department of Neurology, Haaglanden Medical Center, The Hague, the Netherlands;; 12 University NeuroVascular Center (UNVC), Leiden-The Hague, the Netherlands; and; 13 Department of Neurology, University Medical Center Groningen, the Netherlands.

## Abstract

**Background and Objectives:**

Various prehospital scales have been developed to detect patients with anterior-circulation large-vessel occlusion (aLVO) ischemic stroke to enable direct transportation to a thrombectomy-capable stroke center. To guide implementation, a head-to-head comparison of aLVO stroke detection scales is needed to determine which scale is most useful for prehospital triage in different regional contexts. We aimed to systematically identify and compare these scales.

**Methods:**

Published prehospital aLVO stroke scales were identified with a systematic literature search. Scales were reconstructed from individual patient data of 2 large prospective observational cohort studies conducted between 2018 and 2019, the Leiden Prehospital Stroke Study and PREhospital triage of patients with suspected STrOke symptoms study. Both studies included consecutive adult patients suspected by paramedics of having a stroke within 6 hours of symptom onset, from 4 Dutch ambulance regions, encompassing 15 stroke centers and serving 3.7 million people. All data used for the reconstruction of scales were acquired by paramedics in the field before hospital arrival. Scales' diagnostic performance to detect aLVO stroke was compared with the area under the receiver operating characteristic curve (AUROC) of the full scale and sensitivity and specificity at the scales' original cut-point. Decision curve analysis was used to evaluate harm-benefit trade-offs between delaying IV thrombolysis and expediting endovascular thrombectomy with direct transportation of patients to a thrombectomy-capable center.

**Results:**

We identified 63 aLVO scales, of which 14 could be reconstructed. Of 2,358 included patients (mean age 70 years; 47% female), 231 (9.8%) had aLVO stroke. The AUROC was highest for Rapid Arterial oCclusion Evaluation (RACE) (0.81, 95% CI 0.78–0.84), Los Angeles Motor Scale (LAMS) (0.80, 95% CI 0.77–0.83), Gaze-Face-Arm-Speech-Time (G-FAST) (0.80, 95% CI 0.77–0.83), and modified Gaze-Face-Arm-Speech-Time (mG-FAST) (0.79, 95% CI 0.76–0.82). The Emergency Medical Stroke Assessment had highest sensitivity (85%, 95% CI 80%–90%) but lowest specificity (58%, 95% CI 56%–61%) while Cincinnati Prehospital Stroke Scale with an adjusted cut-point of 3 + gaze had highest specificity (94%, 95% CI 93%–95%) but lowest sensitivity (35%, 95% CI 29%–41%). In decision curve analysis, RACE had the highest benefit across a clinically reasonable range of harm-benefit trade-offs.

**Discussion:**

RACE, LAMS, G-FAST, and mG-FAST are the best-performing scales, with RACE being preferred in most triage settings. Our findings may support policymakers with implementing a scale suitable for their region.

## Introduction

In ischemic stroke, the benefit of IV thrombolysis (IVT) and endovascular thrombectomy (EVT) is highly time dependent.^1,2^ EVT is primarily reserved for patients with anterior-circulation intracranial large-vessel occlusion (aLVO), a subgroup known to contribute disproportionately to stroke-related dependence and death.^3^ While IVT is also available in primary stroke centers (PSCs), EVT is restricted to comprehensive or thrombectomy-capable stroke centers (collectively referred to as TSCs). Direct allocation of patients with aLVO stroke to a TSC, bypassing a closer PSC, mitigates delays inherent to interhospital transfers, resulting in faster EVT and better clinical outcomes for these patients.^4-6^ However, bypassing a PSC may also delay other treatments, leading to worse clinical outcomes especially for non-aLVO patients who are eligible for IVT.

To optimize the prehospital triage of patients suspected of having an acute stroke (so-called stroke code patients), various scales have been developed. However, comparative studies of these scales are hampered because of restriction to patients with ischemic stroke rather than all stroke code patients,^7-11^ inclusion of only a limited number of scales,^10-12^ the use of retrospectively collected data,^10,13^ or the use of assessments conducted by physicians in the hospital rather than by paramedics in the prehospital setting.^7-12,14^ Consequently, meta-analyses comparing these scales included heterogeneous populations and showed inconsistent results.^15,16^ To address these gaps, 2 prospective cohort studies compared several aLVO stroke detection scales with assessments conducted by paramedics in stroke code patients.^17,18^ Since the publication of these studies, several novel detection scales have become available. In this article, we aim to (1) systematically identify all published prehospital aLVO stroke detection scales and (2) compare their performance in a pooled analysis with individual patient data from 2 cohorts of stroke code patients.

## Methods

### Systematic Identification of aLVO Stroke Scales

We conducted a systematic literature search until January 2023 in PubMed and EMBASE to identify articles written in English that describe aLVO stroke scales. We used the search terms “stroke,” “prehospital,” “ambulance,” “detection,” “large vessel occlusion,” and “endovascular thrombectomy” with several corresponding synonyms. The full search strategy can be found online in eTable 1. Two reviewers (L.D. and J.D.D.) independently screened titles and abstracts for scales that were based on demographic or clinical features and used a scoring method with a specified cut-point value to detect aLVO stroke in stroke code patients, and obtained full-text versions from studies meeting these criteria. Reference lists were screened for additional studies, and this method was continued until no new publications were found. We excluded scales that could not be reconstructed from the available prehospital data and documented the reasons for this (eTable 2). We followed the STARD reporting guideline for diagnostic accuracy studies.^19^

### Study Design and Population

We used individual patient data from the PREhospital triage of patients with suspected STrOke symptoms (PRESTO) study and the Leiden Prehospital Stroke Study (LPSS).^17,18^ Both were prospective, multiregional, observational cohort studies that included consecutive stroke code patients between July 2018 and October 2019 from 4 Dutch ambulance regions including 10 PSCs and 5 TSCs, serving approximately 3.7 million inhabitants. A stroke code was activated if there was a suspicion of acute stroke, based on a positive Face-Arm-Speech Time (FAST) test or other (focal) neurologic symptoms at the discretion of the individual paramedic.^20^ For this study, we included adult stroke code patients assessed by paramedics within 6 hours of symptom onset or time last seen well, because outside this time window, CT angiography was not always performed. It was routine policy to transport stroke code patients to the nearest stroke center (PSC or TSC).

### Data Collection and Scale Reconstruction

Paramedics routinely documented patient characteristics such as vital parameters, blood glucose, and driving time in electronic transport records. In both studies, a web-based application containing 9 (PRESTO) or 11 (LPSS) structured neurologic deficits was completed by paramedics before hospital arrival. In the PRESTO study, entering all items in the application was mandatory, whereas in LPSS, items could be omitted. Items scored as untestable were set to missing. Electronic patient records from the hospitals were used to extract demographic characteristics, medical history, medication use, admission NIH Stroke Scale (NIHSS) scores,^21^ neuroimaging data, and final diagnosis.

The aLVO stroke scales identified with the systematic literature search were reconstructed from the prehospitally acquired data from the web-based application. The following 9 items were assessed in both studies and used for scale reconstruction: facial palsy, arm motor function, leg motor function, speech disturbances (aphasia or dysarthria), gaze deviation, agnosia (anosognosia/asomatognosia), grip strength, answering questions, and following commands. To optimize uniformity, we marginally amended the scoring methods of some items of Cincinnati Stroke Triage Assessment Tool (C-STAT), Los Angeles Motor Scale (LAMS), and RACE compared with the original PRESTO and LPSS analyses (eMethods 1). In addition, because a pronator drift of the arm was assessed separately in LPSS only, patients derived from LPSS scored points for “any arm weakness” starting from a pronator drift, whereas patients derived from the PRESTO study scored points starting from a mild paresis.

### Outcomes

The primary outcome was a final diagnosis of symptomatic aLVO ischemic stroke, defined as new neurologic deficits corresponding to an occlusion of the internal carotid artery, M1 or M2 segments of the middle cerebral artery, or A1 or A2 segments of the anterior cerebral artery, as assessed on CT angiography. In the PRESTO study, the final diagnosis was made by the treating physician at discharge and the presence of an aLVO was validated by re-assessment of neuroimaging by an imaging core laboratory. Members of the imaging core laboratory were unaware of the diagnosis and prehospital scores but aware of certain clinical symptoms (i.e., side of hemiparesis, aphasia, or nonlocalizing symptoms). In LPSS, the final diagnosis was made by the treating physician after 3 months and aLVO was based on the local assessment of neuroimaging without findings of paramedics being available.

### Statistical Analysis

#### Analysis of Full Scales

We assessed the diagnostic performance of full scales to detect aLVO stroke with the area under the receiver operating characteristic curve (AUROC). The AUROCs of paired receiver operating characteristic curves, that is, in the same population, were compared using the DeLong test.^22^ A 2-sided *p* value of <0.05 was set as significance level, which was adjusted with a post hoc Bonferroni correction for multiple comparisons resulting in an adjusted *p* value of <0.00055 (0.05/91) being considered statistically significant. The AUROC of the in-hospital NIHSS assessed by physicians was reported and plotted as a reference.

#### Analysis of Dichotomized Scales

Because scales are often used as dichotomous tools in clinical practice, we also assessed the diagnostic performance of dichotomized scales at the cut-points as described in the original studies with the sensitivity, specificity, positive predictive value (PPV), negative predictive value (NPV), and the AUROC at the original cut-point (eTable 3). Moreover, we calculated the proportion of EVT-treated patients with aLVO stroke that would have been missed by each scale. In addition, we reported these measures at every possible cut-point of each scale. For diagnostic measures, 95% CIs were calculated by pooling the standard errors of the point estimates with Rubin rules.^23^

#### Decision Curve Analysis

To address the clinical decision-making problem regarding whether stroke code patients should bypass a PSC for a more distant TSC, we performed decision curve analysis.^24^ This analysis explores the net benefit of using each scale across a clinically reasonable range of threshold probabilities, which can be seen as representing different triage settings with different harm-benefit trade-offs.^24-26^ For example, a threshold probability of 20% implies that the benefit of correctly allocating 1 patient with aLVO stroke to a TSC (a true positive), reducing time to EVT, is seen as equivalent to the harm of allocating 4 patients with other diagnoses to a TSC (4 false positives), delaying their treatment in a PSC, such as IVT, blood pressure reduction, or other therapies. This threshold probability varies with the assumed harm-benefit trade-off, which differs between local geographics, logistics, and individual or regional preferences. Therefore, the net benefit is presented as decision curves across a reasonable range of threshold probabilities. The net benefit is defined as the proportion of patients correctly allocated to a TSC minus the proportion of patients incorrectly allocated to a TSC adjusted by a weighting factor, compared with a strategy allocating no patients directly to a TSC. It is calculated as $\text{net benefit}=\frac{\text{true positives}}{\text{total sample size}}-(\frac{\text{false positives}}{\text{total sample size}})\times \text{weighting factor}$. The weighting factor reflects the esteemed value of a true positive to a false positive, depending on the preferred threshold probability ($\text{weighting}\;\text{factor}=\frac{\text{threshold}\;\text{probability}}{\left(1-\text{threshold}\;\text{probability}\right)}$).^24,25^ By plotting the decision curve of each scale, one can determine the strategy that maximizes the net benefit at a specific threshold probability, indicating the highest clinical value for that specific triage setting.

#### Missing Data

For missing data, we used multiple imputation by chained equations with 5 imputations using Rubin rules (eMethods 2).^23,27^ Analyses were performed using R (version 4.3.2) and RStudio (version 2023.09.01+494) with the tidyverse (version 2.0.0), mice (version 3.16.0), pROC (version 1.18.5), and dcurves (version 0.4.0) packages.

### Standard Protocol Approvals, Registrations, and Patient Consents

PRESTO study and LPSS were reviewed by the relevant medical ethical review committees and approved by the institutional review boards of all participating centers. For both studies, the need for obtaining informed consent was waived.

### Data Availability

In compliance with the Dutch law, the individual deidentified patient data cannot be made available because patients were not informed during the opt-out procedure about the public sharing of their individual data. The code and output files of the analyses will be made available on reasonable request from any qualified investigator.

## Results

### Systematic Literature Search

**Figure 1:**
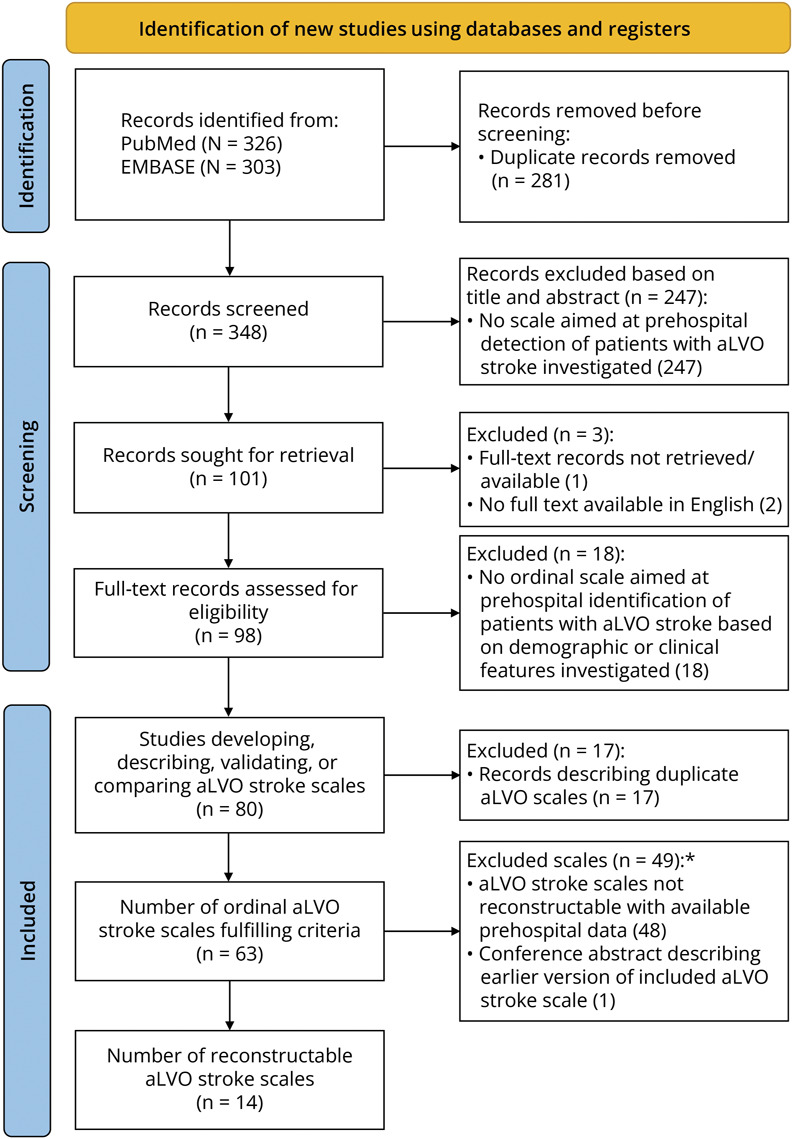
PRISMA Flow Diagram *An overview of excluded aLVO stroke scales and the reasons for their exclusion can be found in eTable 2. aLVO = anterior-circulation large-vessel occlusion; PRISMA = Preferred Reporting Items for Systematic Reviews and Meta-Analyses.

We identified 80 studies describing a total of 63 aLVO stroke detection scales based on clinical features. Of these, 49 were excluded (Preferred Reporting Items for Systematic Reviews and Meta-Analyses flow diagram in Figure 1). The main reasons for exclusion were the more extensive scoring of certain deficits (e.g., different forms of aphasia or categories of motor function loss), the inclusion of specific deficits (e.g., visual field defects or tactile extinction), or medical history (e.g., atrial fibrillation) that was not prehospitally documented in the PRESTO study or LPSS (more details in eTable 2). This left 14 scales for comparison: the Conveniently-Grasped Field Assessment Stroke Triage (CG-FAST),^28^ Cincinnati Prehospital Stroke Scale with an adjusted cut-point of 3 (CPSS3),^29^ CPSS3 combined with gaze deviation (CPSS3 + gaze),^30^ C-STAT (formerly known as CPSSS),^31^ CPSS3 combined with the C-STAT (CPSS + C-STAT),^30^ Emergency Medical Stroke Assessment (EMSA),^32^ Face-Arm-Speech-Time plus severe arm or leg motor deficit test (FAST-PLUS),^33^ Gaze-Face-Arm-Speech-Time (G-FAST),^34^ Gaze Deviation and Paresis Score (GPS),^35^ LAMS,^36^ LAMS combined with speech abnormality (LAMS + speech),^37^ modified Gaze-Face-Arm-Speech-Time (mG-FAST),^38^ Prehospital Acute Stroke Severity,^39^ and Rapid Arterial oCclusion Evaluation (RACE).^40^ More details of these scales are given in eTable 3.

### Baseline Characteristics

**Figure 2:**
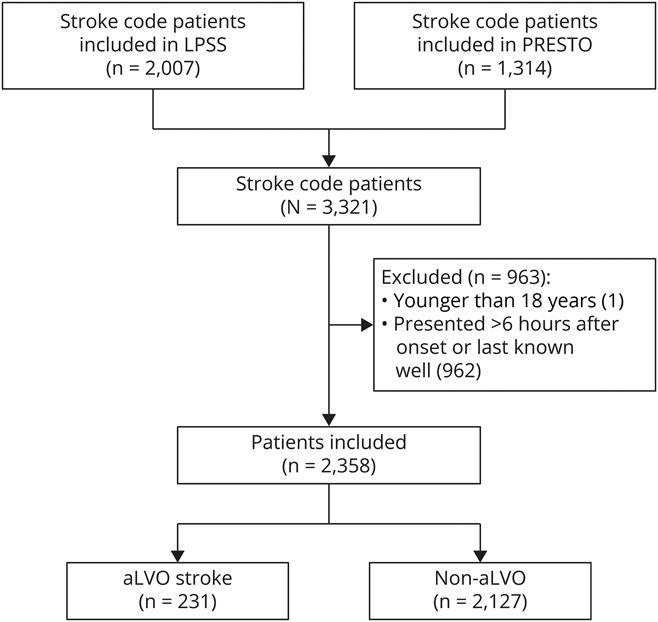
Flowchart of Included Patients aLVO = anterior-circulation large-vessel occlusion; LPSS = Leiden Prehospital Stroke Study; PRESTO = PREhospital triage of patients with suspected STrOke symptoms.

Of the 3,321 stroke code patients enrolled in the PRESTO study and LPSS, 1 patient was younger than 18 years and 962 presented over 6 hours after time of symptom onset or last seen well or at unknown time and were, therefore, excluded (Figure 2). Among the remaining 2,358 patients, the mean age was 70 years (standard deviation 15) and 1,114 (47.2%) were female (Table 1). A total of 1,048 patients (44.4%) presented in a PSC and 1,310 (55.6%) in a TSC. Ischemic stroke was diagnosed in 1,062 (45.0%), with 231 (9.8%) having a symptomatic aLVO. In addition, 174 (7.4%) had an intracranial hemorrhage, 400 (17.0%) a TIA, and 722 (30.6%) a stroke mimic. The median time from symptom onset to ambulance arrival was 55 minutes (interquartile range [IQR] 23–132), the median onset-to-door time was 87 minutes (IQR 54–162), the median door-to-needle time for IVT was 21 minutes (IQR 16–29), and the median door-to-groin time for EVT was 52 minutes (IQR 26–68). In patients with aLVO stroke, the median NIHSS score was 12 (IQR 6–17), and 170 (73.6%) of them underwent EVT, of whom 63 (37.1%) were initially presented at a PSC.

**Table 1:** Characteristics of Included Patients, Stratified per Final Diagnosis

Characteristic	Full cohort (n = 2,358)	aLVO ischemic stroke (n = 231)	Non-aLVO ischemic stroke (n = 831)	Intracranial hemorrhage (n = 174)	TIA (n = 400)	Stroke mimic (n = 722)
Demographic						
Age, y, mean (SD)	70 (15)	72 (12)	72 (14)	72 (14)	73 (13)	66 (16)
Female, n (%)	1,114 (47)	100 (43)	360 (43)	80 (46)	176 (44)	398 (55)
Initial presentation at PSC, n (%)	1,048 (44)	83 (36)	400 (48)	57 (33)	210 (53)	298 (41)
Initial presentation at TSC, n (%)	1,310 (56)	148 (64)	431 (52)	117 (67)	190 (48)	424 (59)
Medical history, n (%)						
Ischemic stroke or TIA^a^	706/2,345 (30)	39/229 (17)	278/828 (34)	31/173 (18)	134/398 (3)	224/717 (31)
Intracranial hemorrhage	63/2,355 (3)	1/229 (0.4)	11/831 (1)	10/174 (6)	12/400 (3)	29/721 (4)
Atrial fibrillation	358/2,346 (15)	45/228 (20)	120/827 (15)	37/174 (21)	72/399 (18)	84/718 (12)
Myocardial infarction	259/2,340 (11)	27/229 (12)	106/824 (13)	14//174 (8)	45/398 (11)	67/715 (9)
Diabetes mellitus	469/2,347 (20)	50/229 (22)	178/827 (22)	21//174 (12)	90/399 (23)	130/718 (18)
Hypercholesterolemia^b^	1,281/2,342 (55)	114/228 (50)	493/826 (60)	85/174 (49)	244/398 (61)	345/716 (48)
Hypertension^b^	1,432/2,346 (61)	142/229 (62)	544/829 (66)	109/174 (63)	265/397 (67)	372/717 (52)
Medication, n (%)						
Antiplatelets	858/2,335 (37)	75/229 (33)	338/826 (41)	46/171 (27)	159/397 (40)	240/712 (34)
Oral anticoagulation	382/2,327 (16)	39/229 (17)	127/822 (15)	35/172 (20)	81/395 (21)	100/709 (14)
Prehospital assessment						
Onset-to-ambulance-arrival time, min, median (IQR)	55 (23–132)	38 (14–127)	62 (25–153)	39 (18–103)	52 (27–107)	61 (27–138)
Systolic blood pressure, mm Hg, median (IQR)	160 (142–183)	149 (137–177)	165 (148–185)	182 (161–204)	160 (143–185)	154 (136–174)
Diastolic blood pressure, mm Hg, median (IQR)	91 (80–103)	88 (78–100)	92 (82–104)	102 (90–117)	91 (80–103)	89 (80–100)
Blood glucose, mmol/L, median (IQR)	6.5 (5.5–7.9)	6.5 (5.6–8.4)	6.5 (5.6–7.9)	6.8 (5.6–8.1)	6.2 (5.4–7.5)	6.4 (5.6–7.8)
In-hospital assessment						
Onset-to-door time, min, median (IQR)	87 (54–162)	70 (45–159)	88 (55–180)	72 (46–144)	84 (56–134)	93 (59–166)
NIHSS score (range 0–42), median (IQR)	2 (0–5)	12 (6–17)	3 (1–5)	11 (4–17)	0 (0–1)	1 (0–3)
Systolic blood pressure, mm Hg, median (IQR)	160 (140–180)	155 (140–173)	164 (146–182)	180 (159–208)	160 (141–181)	152 (134–173)
Diastolic blood pressure, mm Hg, median (IQR)	86 (77–98)	84 (74–93)	87 (76–98)	99 (86–110)	86 (76–98)	84 (76–95)
Blood glucose, mmol/L, median (IQR)	6.6 (5.6–8.0)	6.8 (5.9–8.3)	6.5 (5.6–8.0)	7.3 (6.1–8.6)	6.2 (5.5–7.5)	6.6 (5.7–8.0)
Treatment						
IVT, n (%)	654/2,358 (28)	142/231 (61)	465/831 (56)	0	0	47/722 (7)
Door-to-needle time, minutes, median (IQR)	21 (16–29)	20 (16–29)	22 (16–28)	NA	NA	24 (18–32)
EVT, n (%)	172/2,358	170/231 (74)	2/831 (0.2)^c^	0	0	0
Door-to-groin time, min, median (IQR)	52 (26–68)	52 (26–67)	67 (59–74)	NA	NA	NA
Transfer from PSC to TSC for EVT, n (%)	64/172 (37)	63/170 (37)	1/2 (50)	NA	NA	NA

Abbreviations: aLVO = anterior-circulation large-vessel occlusion; DSA = digital subtraction angiography; eTICI = expanded Thrombolysis In Cerebral Infarction; EVT = endovascular thrombectomy; IQR = interquartile range; IVT = IV thrombolysis; NIHSS = NIH Stroke Scale; PSC = primary stroke center; TSC = thrombectomy-capable stroke center.

a In the PRESTO study, only information on a history of ischemic stroke was collected.

b In LPSS, patients were also considered to have a history of hypercholesterolemia in case of statin use and of hypertension in case of antihypertensive drug use.

c One patient with a basilar artery occlusion also underwent EVT (eTICI 3) for which the patient was transferred from a PSC to TSC. One patient without an occlusion on core laboratory assessment also underwent either DSA or EVT (no further specifications available).

Concerning the data used to reconstruct the aLVO stroke scales, 6.7% of the 9 tested neurologic deficits were missing, with the highest rate for leg motor function (17.6%), agnosia (11.6%), and arm motor function (10.9%).

### Scale Comparison

#### Analysis of Full Scales

**Figure 3:**
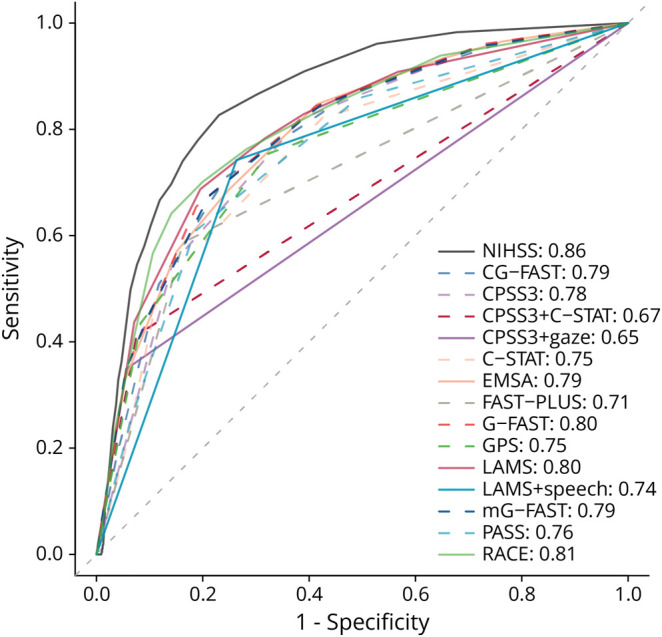
Diagnostic Performance of 14 aLVO Stroke Scales in a Receiver Operating Characteristic Curve The dark gray line depicts the performance of the NIHSS assessment by physicians. Listed are the areas under the curve for each scale. aLVO = anterior-circulation large-vessel occlusion; NIHSS = NIH Stroke Scale.

AUROCs of the full aLVO stroke detection scales ranged from 0.65 to 0.81 (Table 2, Figure 3). The AUROCs of RACE (0.81), LAMS (0.80), G-FAST (0.80), and mG-FAST (0.79) were significantly higher than those of CPSS3 + gaze (0.65), CPSS3 + C-STAT (0.67), FAST-PLUS (0.71), LAMS + speech (0.74), and C-STAT (0.75) (all *p* < 0.00055). Except for FAST-PLUS, all scales had significantly higher AUROCs than CPSS3 + gaze and CPSS3 + C-STAT. AUROCs of other scales ranged from 0.75 to 0.79 and generally did not differ significantly from each other (eTable 4). As a reference, the AUROC of the in-hospital NIHSS was 0.86 (95% CI 0.84–0.89).

**Table 2:** Diagnostic Performance of aLVO Stroke Detection Scales

aLVO stroke scale	AUROC^a^ (95% CI)	Sensitivity, % (95% CI)	Specificity, % (95% CI)	PPV, % (95% CI)	NPV, % (95% CI)	AUROC^b^ (95% CI)	Proportion of missed EVT-treated patients with aLVO stroke, % (95% CI) (n = 170)
CG-FAST	0.79 (0.76–0.82)	51 (44–57)	88 (87–90)	32 (27–37)	94 (93–95)	0.70 (0.66–0.73)	40 (32–47)
CPSS3	0.78 (0.75–0.81)	55 (48–61)	86 (84–87)	29 (25–34)	95 (94–96)	0.70 (0.67–0.74)	39 (31–46)
CPSS3 + C-STAT	0.67 (0.64–0.70)	41 (35–48)	92 (91–94)	37 (31–43)	94 (92–95)	0.67 (0.64–0.70)	51 (43–59)
CPSS3 + gaze	*0.65 (0.61–0.68)*	*35 (29–41)*	**94 (93–95)**	**40 (33–47)**	*93 (92–94)*	*0.65 (0.61–0.68)*	*57 (49–64)*
C-STAT	0.75 (0.72–0.79)	56 (50–63)	82 (80–84)	26 (22–29)	95 (94–96)	0.69 (0.66–0.73)	33 (26–40)
EMSA	0.79 (0.76–0.82)	**85 (80–90)**	*58 (56–61)*	*18 (16–20)*	**97 (96–98)**	0.72 (0.69–0.74)	**9 (4–13)**
FAST-PLUS	0.71 (0.68–0.75)	59 (52–65)	84 (82–85)	28 (24–32)	95 (94–96)	0.71 (0.68–0.75)	33 (26–41)
G-FAST	0.80 (0.77–0.83)	65 (59–72)	81 (80–83)	27 (24–31)	96 (95–97)	0.73 (0.70–0.77)	25 (19–32)
GPS	0.75 (0.72–0.79)	43 (37–50)	92 (90–93)	36 (30–42)	94 (93–95)	0.68 (0.64–0.71)	45 (38–53)
LAMS	0.80 (0.77–0.83)	69 (63–75)	80 (79–82)	28 (24–31)	96 (95–97)	0.75 (0.71–0.78)	24 (18–31)
LAMS + speech	0.74 (0.71–0.77)	74 (68–80)	74 (72–76)	23 (20–27)	96 (95–97)	0.74 (0.71–0.77)	19 (13–25)
mG-FAST	0.79 (0.76–0.82)	67 (61–74)	79 (77–81)	26 (22–29)	96 (95–97)	0.73 (0.70–0.76)	22 (16–29)
PASS	0.76 (0.73–0.79)	61 (54–67)	82 (80–83)	26 (23–30)	95 (94–96)	0.71 (0.68–0.74)	28 (21–34)
RACE	**0.81 (0.78–0.84)**	64 (58–70)	86 (84–88)	33 (29–38)	96 (95–97)	**0.75 (0.72–0.78)**	26 (19–33)

Abbreviations: aLVO = anterior-circulation large-vessel occlusion; AUROC = area under the receiver operating characteristic curve; CG-FAST = Conveniently-Grasped Field Assessment Stroke Triage; CPSS3 = Cincinnati Prehospital Stroke Scale with an adjusted cut-point of 3; C-STAT = Cincinnati Stroke Triage Assessment Tool; CPSS3 + C-STAT = CPSS3 combined with the C-STAT; CPSS3 + gaze = CPSS3 combined with gaze deviation; EMSA = Emergency Medical Stroke Assessment; EVT = endovascular thrombectomy; FAST-PLUS = Face-Arm-Speech-Time plus severe arm or leg motor deficit test; G-FAST = Gaze-Face-Arm-Speech-Time; GPS = Gaze Deviation and Paresis Score; LAMS = Los Angeles Motor Scale; LAMS + speech = LAMS combined with speech abnormality; mG-FAST = modified Gaze-Face-Arm-Speech-Time; NPV = negative predictive value; PASS = Prehospital Acute Stroke Severity; PPV = positive predictive value; RACE = Rapid Arterial oCclusion Evaluation.

The highest value is shown in bold, and the lowest value is shown in italic.

a AUROC for the full range of the aLVO stroke scale.

b AUROC at the original cut-point of the aLVO stroke scale.

#### Analysis of Dichotomized Scales

EMSA had the highest sensitivity (85%, 95% CI 80%–90%) while CPSS3 + gaze (35%, 95% CI 29%–41%) and CPSS3 + C-STAT (41%, 95% CI 35%–48%) had the lowest sensitivity (Table 2). By contrast, specificity was highest for CPSS3 + gaze (94%, 95% CI 93%–95%), GPS (92%, 95% CI 90%–93%), and CPSS3 + C-STAT (92%, 95% CI 91%–94%), whereas it was lowest for EMSA (58%, 95% CI 56%–61%). The PPV ranged from 18% (95% CI 16%–20%) for EMSA to 40% (95% CI 33%–47%) for CPSS3 + gaze. Furthermore, all scales had high NPV, ranging from 93% (95% CI 92%–94%) for CPSS3 + gaze to 97% (95% CI 96%–98%) for EMSA. Finally, EMSA also missed the fewest patients with aLVO stroke treated with EVT (9%, 95% CI 4%–13%) while CPSS3 + gaze had the highest proportion of missed EVT-treated patients (57%, 95% CI 49%–64%). The AUROC at the original single cut-points of scales ranged from 0.65 (95% CI 0.61–0.68) for CPSS3 + gaze to 0.75 for RACE (95% CI 0.72–0.78) and LAMS (95% CI 0.71–0.78). Diagnostic performance metrics at every possible cut-point of the scales are presented in eTable 5.

#### Decision Curve Analysis

**Figure 4:**
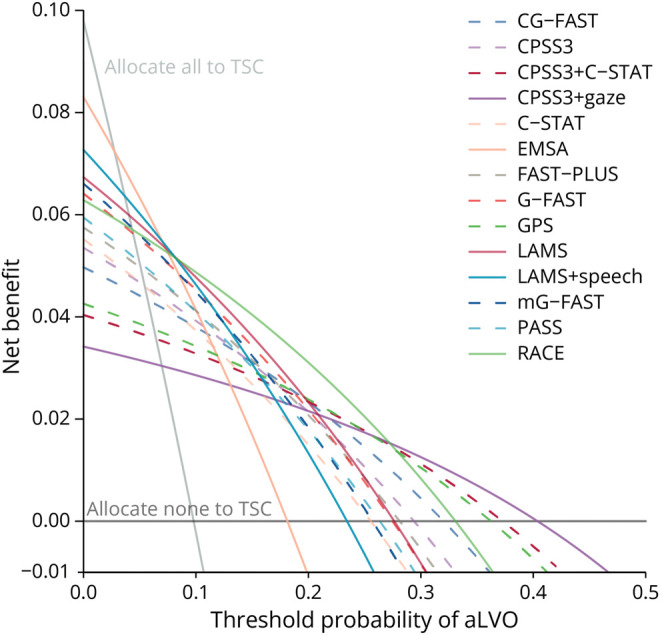
Decision Curve Analysis The odds at the threshold probability (x-axis) reflect the trade-off between the benefit of correctly allocating 1 patient with aLVO stroke against the harm of allocating a certain number of patients with other diagnoses to a TSC. The net benefit (y-axis) is the net increase in the proportion of patients with aLVO stroke who are correctly allocated to a TSC, compared with the strategy allocating no patients directly to a TSC. At each specific threshold, the strategy with the highest net benefit is preferred. aLVO = anterior-circulation large-vessel occlusion; TSC = thrombectomy-capable center.

Decision curve analysis showed that directly allocating all patients to a TSC was most beneficial up to a threshold probability of 3% (Figure 4, eMethods 3). Subsequently, EMSA had the highest net benefit until a threshold probability of 7%. RACE was preferred over the widest range of thresholds (8%–27%). Finally, CPSS3 + gaze performed best up to 40%. For higher threshold probabilities, allocating all patients to the closest stroke center without additional triage was most beneficial.

## Discussion

Through a systematic literature search, we identified 63 aLVO stroke detection scales, of which 14 could be reconstructed and validated with prospectively acquired prehospital data. Among these scales, RACE, LAMS, G-FAST, and mG-FAST had the highest diagnostic performance to detect aLVO stroke within a stroke code population. RACE showed the highest clinical value in most triage settings and trade-offs. Furthermore, EMSA exhibited the highest sensitivity but the lowest specificity while CPSS3 + gaze offered the highest specificity but the lowest sensitivity.

Comparing our findings with previous studies on aLVO stroke scales is challenging because of differences in methodologies.^15,16^ Although current international consensus statements advise the use of a prehospital aLVO stroke detection scale, no specific scale is recommended.^41,42^ In this study, we were able to reconstruct and compare a large number of aLVO stroke scales with individual patient data assessed by paramedics in the field. Our results could assist health care providers and policymakers in making a well-informed decision concerning which scale best fits their situation, given a specific threshold taking into account the local context and available resources. For example, urban regions with sufficient resources may prioritize a scale with a high sensitivity, because delays to IVT that result from bypassing a closer PSC will be relatively short, whereas delays to EVT related to interhospital transfers are relatively long. By contrast, in regions with larger distances or limited resources, a higher specificity may be preferred, because of the longer delays to IVT associated with bypassing a PSC and the additional burden on paramedic and hospital facilities.^43^ Our decision curve analysis indicated that RACE is preferred in most triage settings. However, in rural areas, policymakers could select a higher threshold probability, indicating that for each correct allocation of a patient with aLVO, few false-positive allocations for patients with other diagnoses to a TSC are accepted. For example, for threshold probabilities between 27% and 40%, CPSS3 + gaze is most beneficial, while allocating all patients to the closest stroke center is favored in case of even higher threshold probabilities. By contrast, at very low thresholds, indicating that, to correctly present 1 patient with aLVO directly to a TSC, more patients with other diagnoses unnecessarily bypassing a PSC are accepted, EMSA or simply allocating all patients to the TSC is preferred.

Although some scales performed significantly worse, the AUROC analyses showed only minor, nonsignificant differences among the best-performing scales. Notably, the AUROC analysis using the full scales may favor scales with an increasing number of items because the area under the curve is calculated more gradually than in a dichotomized scale. This likely explains the poorer performance of the 4 scales with the lowest full-scale AUROC because these ultimately yield only a binary positive or negative score. Of interest, in the dichotomous analysis using the scales' specified cut-points, the CG-FAST performed considerably worse while the LAMS + speech performed relatively better than in the full-scale analysis. Although the RACE remained the scale with the highest AUROC, no single scale significantly outperformed the others. However, although AUROC is a useful metric, it does not fully reflect a scale's clinical utility, and the decision curve analysis provides a more comprehensive evaluation of diagnostic qualities. Furthermore, a key clinical consideration is how the inclusion of specific neurologic deficits influences a scale's performance. For example, some scales emphasize speech or cortical signs more prominently while others rely more on motor function. These variations may partly explain differences in performance and warrant further investigation.

The large number of published aLVO stroke detection scales that all rely on clinical deficits with only modest differences in diagnostic performance suggests that the development of new scales may only be justified if they incorporate novel detection methods such as biomarkers, telemedicine, dry-electrode electro-encephalography, or transcranial Doppler ultrasound.^44^ This holds especially true if EVT proves to be beneficial for more distal vessel occlusions because these patients have milder neurologic deficits, which could limit the efficacy of triage scales based on clinical observations.^45,46^ In addition, incorporating patient-specific and region-specific characteristics, such as time since symptom onset, driving and stroke center workflow times, and estimated time-dependent treatment effects of IVT and EVT, could also help improve stroke code allocation.^47^

Strengths of this study include the systematic identification of all published aLVO stroke scales and their evaluation with individual patient data acquired by paramedics in the prehospital setting from 2 large multiregional cohorts of stroke code patients. This allowed for a head-to-head comparison of a substantial number of aLVO stroke scales. Owing to the large sample size and minimal amount of missing data, we were able to evaluate several diagnostic performance metrics and provide precise estimates. In addition, by analyzing both the full-range and dichotomous performance of scales, we provide insights into the performance of scales over the full range of cut-points and when implemented as a dichotomous tool. Finally, the decision curve analysis provides valuable information on the clinical value of a scale as a dichotomous tool in different triage settings.

Our study has some limitations. First, not all aLVO stroke scales could be reconstructed. However, most of these scales rely on deficits similar to those of included scales. Therefore, we do not expect major differences in diagnostic performance of excluded scales. In addition, assessment by paramedics should be straightforward, and more extensive evaluation of neurologic deficits might reduce feasibility in a prehospital setting. Second, the diagnostic performance of scales may vary across different populations and health care systems. Our study was conducted in the Netherlands, where stroke care is highly organized, with relatively short distances between PSCs and TSCs. Furthermore, the use of prehospital triage tools and training of paramedics in our study are specific to the Dutch health care system. These regional characteristics should be considered when applying our findings to other populations. Nonetheless, many regions worldwide may already have incorporated an aLVO detection scale in routine prehospital practice in accordance with current guidelines,^41,42^ and given the similarities in composition of different scales, we expect that paramedics should generally be proficient in their application. Moreover, while variations in paramedic training might influence the absolute diagnostic performance of scales, we found no evidence that this would differentially affect scales, making it unlikely that it would substantially alter our comparative findings. Third, we excluded patients in whom time from symptom onset or last seen well to presentation was over 6 hours or unknown, which may limit the generalizability of our findings to this group of patients. However, there is no reason to assume that the diagnostic performance of scales would differ in patients presenting in this later time window. Fourth, on a patient level, aLVO stroke scales only aid in deciding the optimal transportation strategy for those who are closest to a PSC because patients with stroke closest to a TSC will always benefit most from direct transportation to that TSC. Finally, aside from patients with aLVO stroke, other patients with stroke may also benefit from direct transportation to a TSC, such as patients with basilar artery occlusion or patients with primary intracerebral hemorrhage.^48-50^ Future research should assess the diagnostic performance of scales for these patients.

We identified 63 prehospital aLVO stroke detection scales, of which 14 could be reconstructed from prehospital data. RACE, LAMS, G-FAST, and mG-FAST were the best-performing scales, and RACE was preferred in most triage settings. Our findings may help policymakers to decide on which aLVO stroke scale is most suitable for their region.

## Appendix Coinvestigators

**Table:**

Coinvestigators are listed in the Supplementary Material at Neurology.org/N

## Glossary

aLVO: anterior-circulation large-vessel occlusion
AUROC: area under the receiver operating characteristic curve
CG-FAST: Conveniently-Grasped Field Assessment Stroke Triage
CPSS3: Cincinnati Prehospital Stroke Scale with an adjusted cut-point of 3
CPSS3 + C-STAT: CPSS3 combined with the C-STAT
CPSS3 + gaze: CPSS3 combined with gaze deviation
C-STAT: Cincinnati Stroke Triage Assessment Tool
EMSA: Emergency Medical Stroke Assessment
EVT: endovascular thrombectomy
FAST-PLUS: Face-Arm-Speech-Time plus severe arm or leg motor deficit test
G-FAST: Gaze-Face-Arm-Speech-Time
GPS: Gaze Deviation and Paresis Score
IQR: interquartile range
IVT: IV thrombolysis
LAMS: Los Angeles Motor Scale
LAMS + speech: LAMS combined with speech abnormality
LPSS: Leiden Prehospital Stroke Study
mG-FAST: modified Gaze-Face-Arm-Speech-Time
NIHSS: NIH Stroke Scale
NPV: negative predictive value
PPV: positive predictive value
PRESTO: PREhospital triage of patients with suspected STrOke symptoms
PSC: primary stroke center
RACE: Rapid Arterial oCclusion Evaluation
TSC: thrombectomy-capable stroke center
